# Heterogeneous network promotes species coexistence: metapopulation model for rock-paper-scissors game

**DOI:** 10.1038/s41598-018-25353-4

**Published:** 2018-05-04

**Authors:** Takashi Nagatani, Genki Ichinose, Kei-ichi Tainaka

**Affiliations:** 1 Department of Mechanical Engineering, Shizuoka University, Hamamatsu, 432-8561 Japan; 2 Department of Mathematical and Systems Engineering, Shizuoka University, Hamamatsu, 432-8561 Japan; 3 Graduate School of Science and Technology, Shizuoka University, Hamamatsu, 432-8561 Japan

## Abstract

Understanding mechanisms of biodiversity has been a central question in ecology. The coexistence of three species in rock-paper-scissors (RPS) systems are discussed by many authors; however, the relation between coexistence and network structure is rarely discussed. Here we present a metapopulation model for RPS game. The total population is assumed to consist of three subpopulations (nodes). Each individual migrates by random walk; the destination of migration is randomly determined. From reaction-migration equations, we obtain the population dynamics. It is found that the dynamic highly depends on network structures. When a network is homogeneous, the dynamics are neutrally stable: each node has a periodic solution, and the oscillations synchronize in all nodes. However, when a network is heterogeneous, the dynamics approach stable focus and all nodes reach equilibriums with different densities. Hence, the heterogeneity of the network promotes biodiversity.

## Introduction

Coexistence of multiple species is observed in nature. The mechanism of coexistence has been discussed by many authors^1–4^. Plausible mechanisms for the coexistence have been presented, such as spatial and temporal segregations^5–8^, cooperative interactions^4,9–12^ and so on. In the present paper, we focus on the biodiversity in rock-paper-scissors (RPS) systems^13–21^. We apply metapopulation dynamics^22–24^ to RPS systems, and report that the heterogeneity of a metapopulation network promotes the coexistence of species.

The cyclic balance in ecosystems maintains biodiversity. An example is a relationship among plant, herbivore and carnivore. Herbivores eat plants, but they are eaten by carnivores; when the carnivores die, they become nutrition for plants. Such a cyclic association is very common in real ecosystems^25–30^. More concrete examples of RPS games are the mating strategies of side-blotched lizards^31^, marine sessile organisms^32,33^, mutant strains of yeast^34^, grass-tree systems^35^, three strains of Escherichia coli^36,37^ and fish in fresh water^38^. These species in cyclic relation can coexist in nature.

Much literature exists for theoretical works on the population dynamics in RPS systems. In 1973, Itoh has presented a “well-mixed” (“global interaction”) model for rock (R), scissors (S) and paper (P)^13^. He mathematically proved that the population dynamics are represented by classical Lotka-Volterra equation: the densities of three species (R, S, P) oscillate periodically (“neutrally stable”). However, when the total population size is finite, three species cannot coexist. In contrast, Tainaka has presented a lattice model (stochastic cellular automaton) for RPS games^5,6^. The collision occurs between adjacent sites (“local interaction”). He showed that three species can stably coexist. Such a difference between global and local interactions has been verified by the experiments with Escherichia coli^36^. In the case of local interaction, the population dynamics are largely affected by spatial pattern formation of species^14,20,39,40^.

The spatial RPS game has been extended to network models^41,42^. In these cases, each node means an individual (agent), and a link means the interaction between agents. It is, however, very rare to discuss the relationship between network structure and biodiversity. Masuda and Konno have studied RPS games on complex networks, and discussed the relation between network structure and species coexistence^42^. In the present paper, we discuss the same relation, applying a metapopulation model^22^.

The metapopulation model is popular in biology (ecology)^22–24^. The metapopulation consists of spatially separated habitats (patches or nodes); this is because the whole population of a species is usually separated into some nodes. Individuals can migrate between nodes. In most cases, the individuals move from higher- to lower-density nodes^43–49^. However, in the present model, we apply a “random migration”: each agent randomly determines the destination of migration^50^. The RPS reactions only occur inside each node. By solving the reaction-migration equations analytically or numerically, we show that the RPS dynamics between homogeneous and heterogeneous graphs are significantly different. It is found that three species can stably coexist only on the heterogeneous graph. The heterogeneity can help to maintain the coexistence of species.

## Models

In RPS game, each individual is either rock (R), scissors (S) or paper (P). Interactions (RPS games) take place inside each node as follows:

1a $${\rm{R}}+{\rm{S}}\to {\rm{R}}+{\rm{R}}\,({\rm{rate}}\,a),$$

1b $${\rm{S}}+{\rm{P}}\to {\rm{S}}+{\rm{S}}\,({\rm{rate}}\,b),$$

1c $${\rm{P}}+{\rm{R}}\to {\rm{P}}+{\rm{P}}\,({\rm{rate}}\,c),$$

where the parameters *a*, *b*, and *c* are victory rates^17,40,51^. If $a=b=c=1$, then the system (3) becomes “standard” RPS system^5,6,13^. Three reactions represent a cyclic relation: species R beats S, S beats P, and P beats R.

First, we consider a case of no migration where the total population lives in a single habitat (node)^13,15,38^. When all individuals are assumed to be completely mixed (global interaction), the dynamics for system (1) are given by

2a $$\frac{d{\rho }_{R}(t)}{dt}=a{\rho }_{R}(t){\rho }_{S}(t)-c{\rho }_{P}(t){\rho }_{R}(t),$$

2b $$\frac{d{\rho }_{P}(t)}{dt}=c{\rho }_{P}(t){\rho }_{R}(t)-b{\rho }_{S}(t){\rho }_{P}(t),$$

2c $$\frac{d{\rho }_{S}(t)}{dt}=b{\rho }_{S}(t){\rho }_{P}(t)-a{\rho }_{R}(t){\rho }_{S}(t),$$

where ${\rho }_{\alpha }(t)$ is the density of species *α* at time *t* (*α* = R, S, P). The total population size is assumed to be constant; thus, we put ${\rho }_{R}(t)+{\rho }_{P}(t)+{\rho }_{S}(t)=1$. In the equilibrium state, we set $d{\rho }_{\alpha }/dt=0$. Therefore, the equilibrium densities (non-zero values) are given by

3 $${\rho }_{R,e}=b/(a+b+c),{\rho }_{P,e}=a/(a+b+c),{\rho }_{S,e}=c/(a+b+c).$$

If $a=b=c=1$ (standard RPS game), then all densities take the same value (1/3). The equilibrium state is not stable^37^. The density of each species periodically oscillates around the equilibrium density. The time average of a species density over one period is given by equation (3).

**Figure 1:**
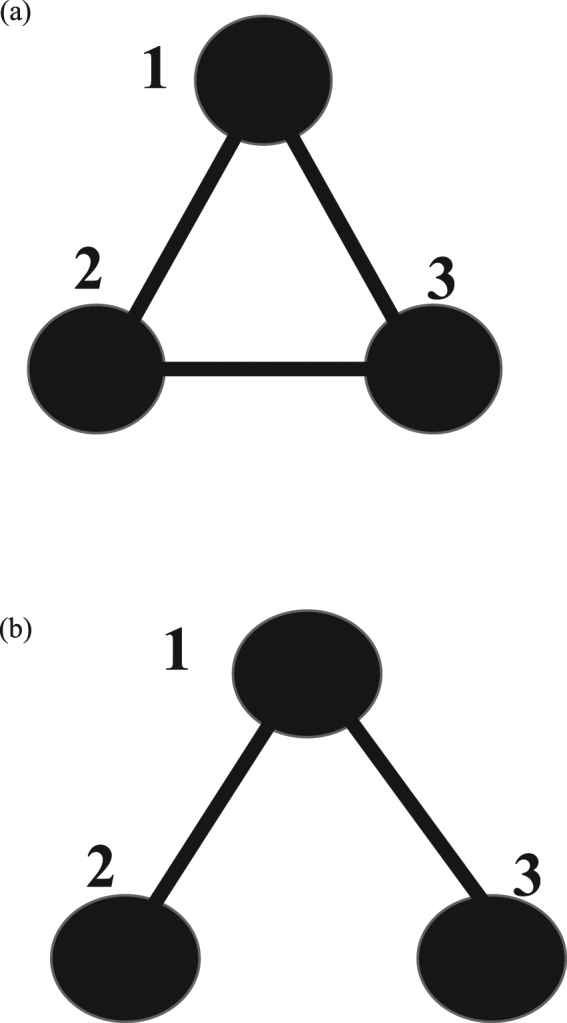
Schematic illustration of the metapopulation model (*N* = 3). Each graph is composed of three subpopulations (nodes) 1, 2, and 3. Each line connecting a pair of nodes denotes the path (link). Two configurations are possible for *N* = 3. (**a**) Homogeneous graph: all nodes have the same degree (number of links). (**b**) Heterogeneous graph.

Next, we consider metapopulation models which have *N* nodes ($N\ge 2$). Population dynamics are expected to be different whether the network is homogeneous or heterogeneous. When all nodes have the same degree (number of links), we call it homogeneous. We choose *N* = 3, because it is the simplest case to have both homogeneous and heterogeneous graphs. In Fig. 1, three circles mean habitats (nodes), and lines denote paths (links). The whole population consists of three nodes (*N* = 3). All individuals can move their nodes along a path. Let ${\rho }_{\alpha i}$ be the density of species *α* in node *i* (*α* = R, S, P and $i=1,2,3$), and ${\rho }_{i}$ be the total density in node *i*. From the definition, we have

4 $${\rho }_{i}=\,{\rho }_{Ri}(t)+{\rho }_{Pi}(t)+{\rho }_{Si}(t)$$

and $\sum _{i}{\rho }_{i}=1$. Provided that the migration rate for all individuals take the same value (unity), the density in node *i* is described by

5 $$d{\rho }_{i}(t)/dt=\sum _{j\in {N}_{i}}(\frac{1}{{k}_{j}}{\rho }_{j}(t)-\frac{1}{{k}_{i}}{\rho }_{i}(t)),$$

where *k*_*i*_ is the degree of node *i* and *N*_*i*_ represents the nearest neighbors of node *i* (sum over all paths)^50^. In equation (5), the terms on the right-hand side indicate the amount of incoming and outgoing individuals. According to mean-field theory, the densities of R, S, and P walkers in node *i* are described by

6a $$d{\rho }_{R,i}(t)/dt=\sum _{j\in {N}_{i}}(\frac{1}{{k}_{j}}{\rho }_{R,j}(t)-\frac{1}{{k}_{i}}{\rho }_{R,i}(t))+[a{\rho }_{R,i}(t){\rho }_{S,i}(t)-c{\rho }_{P,i}(t){\rho }_{R,i}(t)],$$

6b $$d{\rho }_{S,i}(t)/dt=\sum _{j\in {N}_{i}}(\frac{1}{{k}_{j}}{\rho }_{S,j}(t)-\frac{1}{{k}_{i}}{\rho }_{S,i}(t))+[b{\rho }_{S,i}(t){\rho }_{P,i}(t)-a{\rho }_{R,i}(t){\rho }_{S,i}(t)],$$

6c $$d{\rho }_{P,i}(t)/dt=\sum _{j\in {N}_{i}}(\frac{1}{{k}_{j}}{\rho }_{P,j}(t)-\frac{1}{{k}_{i}}{\rho }_{P,i}(t))+[c{\rho }_{P,i}(t){\rho }_{R,i}(t)-b{\rho }_{S,i}(t){\rho }_{P,i}(t)].$$

In equations (6a–c), the first and second terms on the right-hand side represent the migration and reactions of RPS games, respectively.

## Results

### Case of homogeneous networks

**Figure 2:**
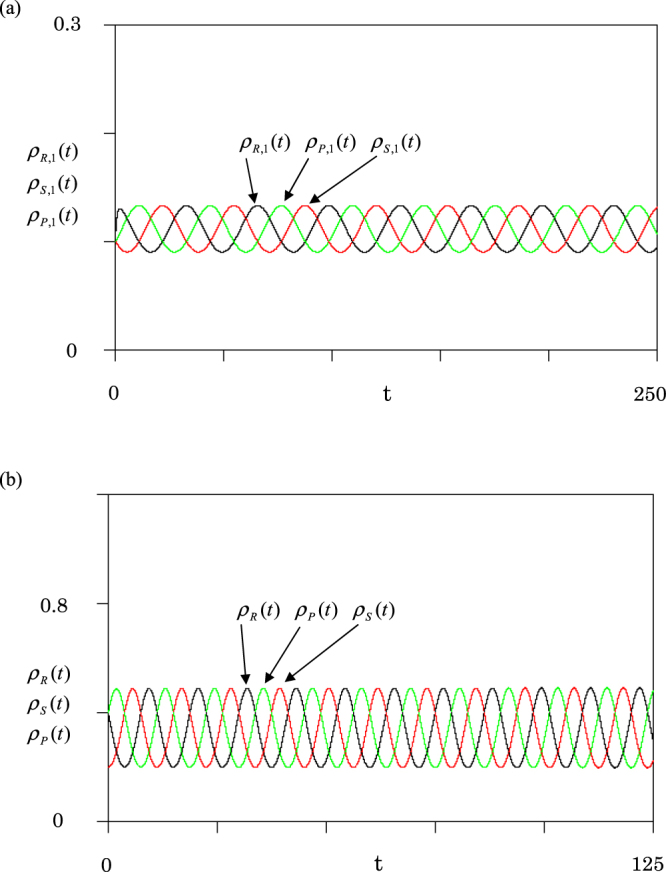
Results of population dynamics at rate $a=b=c=1$. (**a**) Metapopulation model for homogeneous graph in Fig. 1(a). The densities of three species (R, S, P) in node 1 are plotted against time *t*. The initial values are ${\rho }_{\alpha i}(0)=0.1$ for species *α* in node *i* (*α* = R, S, P and $i=1,2,3$), but the rock density in node 3 is only set as ${\rho }_{R,3}(0)=0.2$. Black, red and green curves indicate the densities of rock, scissors, and paper in node 1, respectively. (**b**) A single habitat case without migration. By the use of equation (2), the mean-field dynamics are displayed with initial values ${\rho }_{R}(0)={\rho }_{P}(0)=0.4$ and ${\rho }_{S}(0)=0.2$.

Basic equation (6) is applied to two graphs in Fig. 1 (see Supplementary Materials). First, we report the results for the homogeneous graph [Fig. 1(a)]. The victory rates are set to be equal ($a=b=c=1$: standard RPS game). Figure 2(a) shows the plots of R, S, P densities in node 1 against time *t*. Black, red and green curves indicate the densities of rock, scissors, and paper in node 1, respectively. The density of each species oscillates periodically around the equilibrium density (${\rho }_{\alpha ,i,e}=1/9$). This value is the same for any species *α* and any node *i* (*α* = R, S, P and $i=1,2,3$). For the sake of comparison, Fig. 2(b) displays the population dynamics obtained by equation (2) (no migration case). By the comparison between Fig. 2(a) and (b), we find the frequencies of oscillations in Fig. 2(b) are three times as large as those in Fig. 2(a). In the metapopulation model, the oscillation in each node becomes slower due to the random walk.

**Figure 3:**
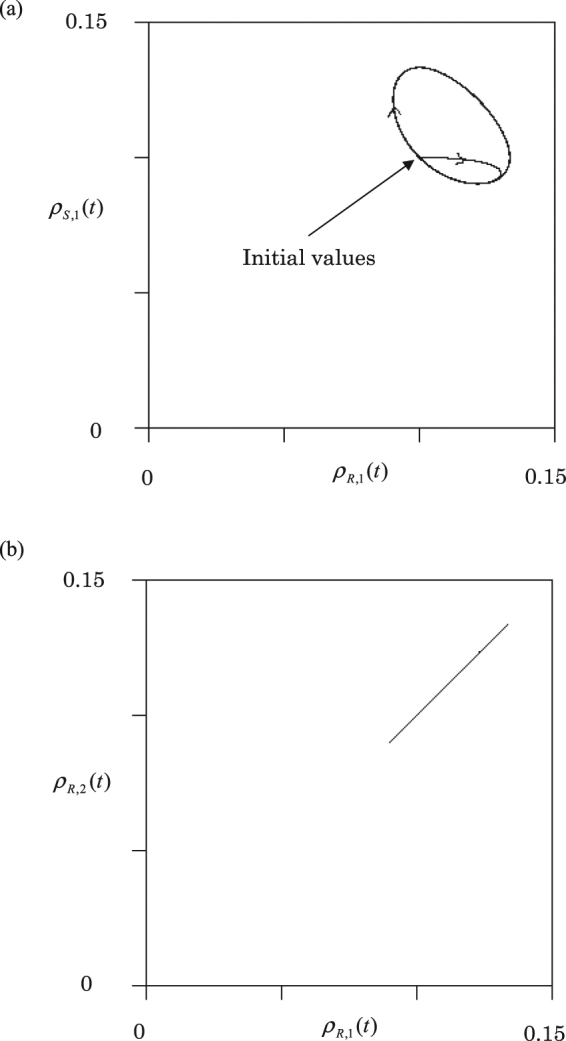
The orbits for homogeneous graph. (**a**) The scissors density ${\rho }_{S,1}(t)$ is plotted against the rock density ${\rho }_{R,1}(t)$ in node 1. (**b**) Plots of rock density ${\rho }_{R,2}(t)$ in node 2 against the rock density ${\rho }_{R,1}(t)$ in node 1.

Figure 3(a) shows an orbit for Fig. 2(a), where the scissors density ${\rho }_{S,1}(t)$ is plotted against rock density ${\rho }_{R,1}(t)$ in node 1. The dynamics finally approach a circle. The orbits rotate clockwise on the circle. The phase difference between rock and scissors densities is just $3\pi /4$. Figure 3(b) shows the plot of ${\rho }_{R,2}(t)$ in node 2 against ${\rho }_{R,1}(t)$ in node 1. When ${\rho }_{R,2}(t)$ increases (decreases), then ${\rho }_{R,1}(t)$ increases (decreases) simultaneously. Hence, the oscillations of rock densities are completely synchronized in both nodes 1 and 2. We can confirm that the densities of each species (R, S, or P) simultaneously oscillate in different nodes.

### Case of heterogeneous networks

**Figure 4:**
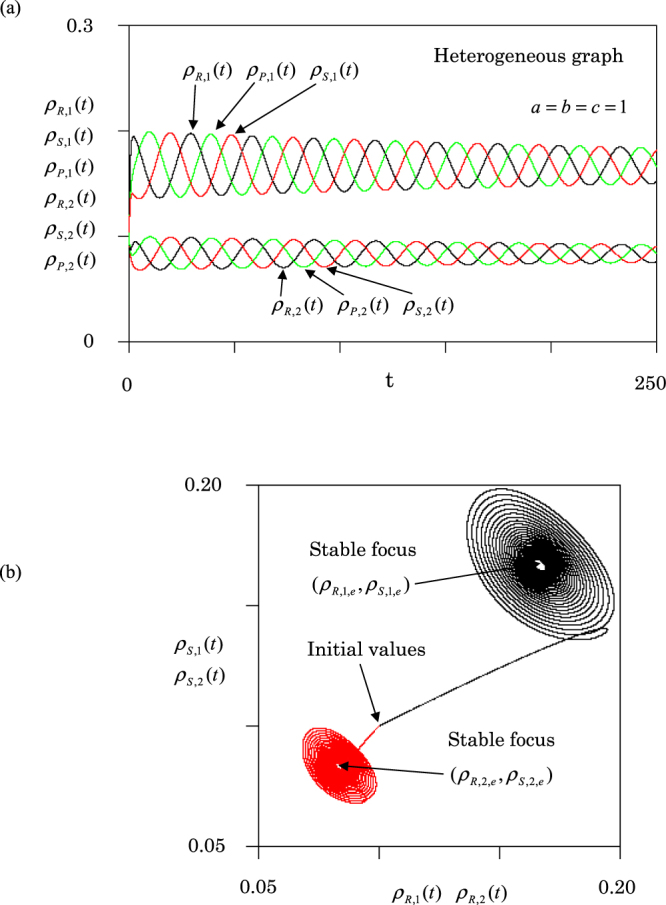
Results of population dynamics for heterogeneous graph [Fig. 1(b)] at rate $a=b=c=1$. (**a**) Three (R, S, P) densities in nodes 1 and 2 are depicted against time *t*. (**b**) Two orbits are displayed. (i) the black orbit: (${\rho }_{S,1}(t)$, ${\rho }_{R,1}(t)$) in node 1, and (ii) the red orbit: (${\rho }_{S,2}(t)$, ${\rho }_{R,2}(t)$) in node 2. Initial values are the same as used in Fig. 2(a).

Next, we report the results for the heterogeneous graph [Fig. 1(b)]. Individuals can migrate between a pair of nodes by random walks. However, they never migrate between nodes 2 and 3. Figure 4(a) shows the population dynamics for the heterogeneous graph. The densities of three species exhibit damping oscillations. The amplitudes are gradually decreased. The densities approach different equilibrium values depending on different nodes:

7 $${\rho }_{\alpha ,1,e}=1/6,{\rho }_{\alpha ,2,e}={\rho }_{\alpha ,3,e}=1/12$$

where $\alpha$ = R, S, or P (see Supplementary Materials). From equation (5), the equilibrium density in each node is given by

8 $${\rho }_{1,e}=1/2,{\rho }_{2,e}={\rho }_{3,e}=1/4.$$

Namely, the density in node 1 is twice as large than that in node 2 (3). Individuals come together in node 1 (hub) by random walk.

In Fig. 4(b), two orbits are shown. i) the black orbit of (${\rho }_{S,1}(t)$, ${\rho }_{R,1}(t)$) in node 1, and ii) the red orbit of (${\rho }_{S,2}(t)$, ${\rho }_{R,2}(t)$) in node 2. Both orbits rotate clockwise and approach equilibrium points (stable focuses). Similarly, the densities in node 3 approach the same equilibrium as in node 2. The stable densities in node 1 and 2 (3) are given by equation (7). Thus, the dynamic behavior on the heterogeneous graph [see Fig. 3(a)] is significantly different from that on the homogeneous graph [see Fig. 4(b)].

### Effect of the victory rates

**Figure 5:**
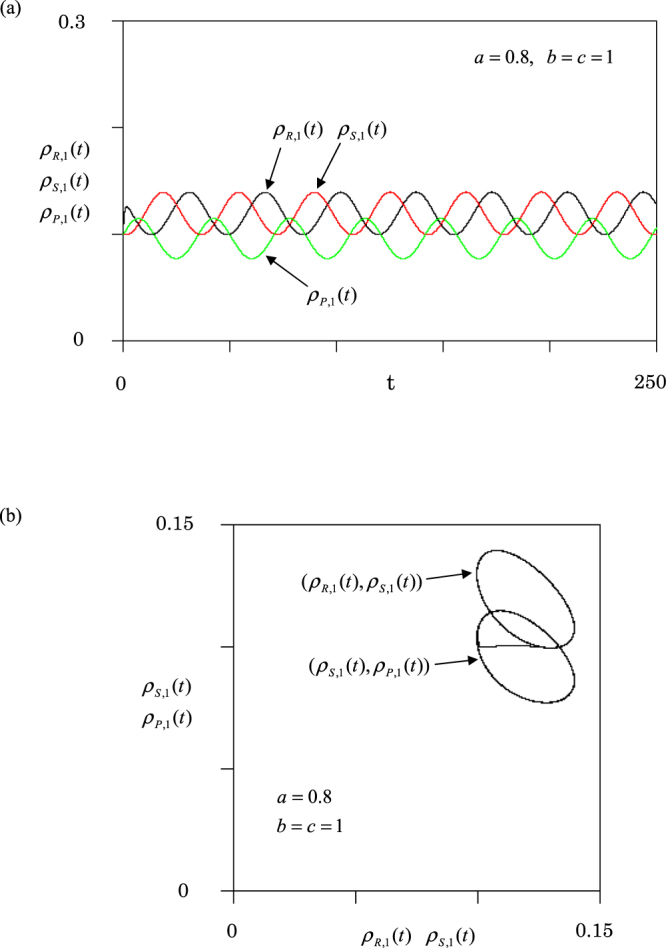
Population dynamics at rate $a=0.8$ and $b=c=1$ for homogeneous graph. (**a**) Three densities in node 1 are plotted against time *t*. (**b**) Two orbits (${\rho }_{R,1}(t)$, ${\rho }_{S,1}(t)$) and (${\rho }_{S,1}(t)$, ${\rho }_{P,1}(t)$) in node 1 are displayed.

In general, the victory rates take arbitrary values. When three rates *a*, *b* and *c* are all changed, the dynamics become very complicated. According to the previous works^17,38,51^ we fix *b* = *c* = 1 and change the value of *a* which is the victory rate of rock. First, we report the results for the homogeneous graph [Fig. 1(a)]. Figure 5 displays the effect of the victory rate, where (a) population dynamics and (b) orbits. The densities oscillate periodically around the equilibrium point expressed by

9 $${\rho }_{R,i,e}={\rho }_{S,i,e}=1/[3(2+a)],\,{\rho }_{P,i,e}=a/[3(2+a)],$$

for any node ($i=1,2,3$) (see Supplementary Materials). Namely, these values are just one-third of equation (3). In Fig. 5(b), two orbits (${\rho }_{R,1}(t)$, ${\rho }_{S,1}(t)$) and (${\rho }_{S,1}(t)$, ${\rho }_{P,1}(t)$) in node 1 are displayed. The final trajectories exhibit two circles where the upper and lower circles represent (${\rho }_{R,1}(t)$, ${\rho }_{S,1}(t)$) and (${\rho }_{S,1}(t)$, ${\rho }_{P,1}(t)$), respectively. The phase difference between rock (scissors) and scissors (paper) is $3\pi /4$. The R, S or P densities synchronize among different nodes. Since we set $a=0.8,$ the average densities of all species over one period are not equal; the densities of paper are lower than those of rocks or scissors in all nodes.

**Figure 6:**
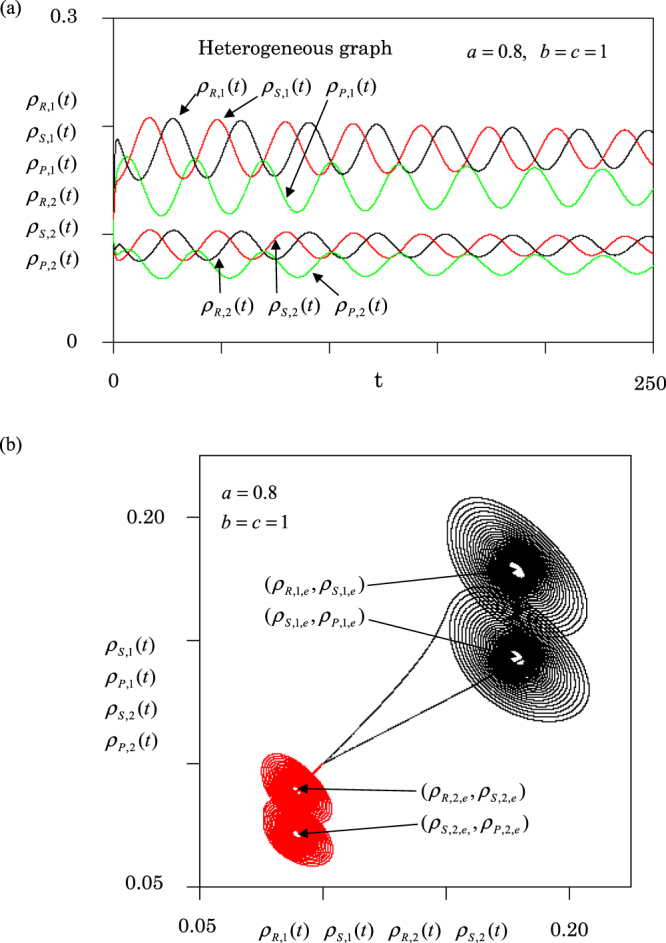
Same as Fig. 5, but for heterogeneous graph. (**a**) Population dynamics. Upper three curves denote the three densities in node 1, while lower three curves mean those in node 2. (**b**) Four orbits. Black curves denote orbits of (*ρ*_*R*,1_ (*t*), ${\rho }_{S,1}(t)$)) and (${\rho }_{S,1}(t)$, ${\rho }_{P,1}(t)$) in node 1, and red curves denote the orbits (${\rho }_{R,2}(t)$, ${\rho }_{S,2}(t)$)) and (${\rho }_{S,2}(t)$, ${\rho }_{P,2}(t)$) in node 2.

Next, we report the results for the heterogeneous graph [Fig. 1(b)]. Figure 6 displays (a) population dynamics and (b) orbits. The upper three curves in Fig. 6(a) show the time dependence of three densities in node 1. The lower three curves represent the densities in node 2 (3). In node 1, three densities approach

10a $${\rho }_{R,1,e}={\rho }_{S,1,e}=1/[2(2+a)],{\rho }_{P,1,e}=a/[2(2+a)].$$

On the other hand, in node 2 (3), they approach

10b $${\rho }_{R,2(3),e}={\rho }_{S,2(3),e}=1/[4(2+a)],{\rho }_{P,2(3),e}=a/[4(2+a)],$$

Figure 6(b) shows the four orbits which exhibit stable focuses. Here, the black curves are orbits of (${\rho }_{R,1}(t)$, ${\rho }_{S,1}(t)$)) and (${\rho }_{S,1}(t)$, ${\rho }_{P,1}(t)$) in node 1, and red curves denote the same orbits but in node 2 (3). Thus, the equilibrium densities expressed by equation (10) are stable on the heterogeneous graph.

## Conclusion

We have developed the metapopulation model for RPS games with three subpopulations (nodes). In metapopulation models, the individuals usually migrate from higher- to lower-density nodes^25,26,40–46^. However, in the present paper, we apply a random migration: each individual randomly determines the destination of migration^50^. The RPS reactions only occur inside each node. By solving the reaction-migration equations analytically or numerically, we show that the RPS dynamics between homogeneous [Fig. 1(a)] and heterogeneous [Fig. 1(b)] graphs are significantly different.

We first show the population dynamics for a two-node graph (*N* = 2). In this case, the dynamics are similar to the single-node case (*N* = 1): the system never approaches an equilibrium state (periodic oscillation). The two-node graph is homogeneous, because both nodes have the same degree (one link). Next, we choose *N* = 3, because it is the simplest case to have both homogeneous and heterogeneous graphs (see Fig. 1). In the case of the homogeneous graph [Fig. 1(a)], the dynamics are similar to the single-node case (see Figs 2, 3 and 5). The amplitudes of oscillations depend on the initial condition, and the frequency becomes one-third compared to the single-node case (see Fig. 2). However, in the case of the heterogeneous graph [Fig. 1(b)], the dynamics are represented by the stable focus (see Figs 4 and 6). Three species (R, P and S) stably coexist. Hence, we conclude that the heterogeneity of networks promotes stable coexistence in RPS systems. We can confirm this conclusion even for the star graph of *N* = 4; the star graph is also heterogeneous, because it has a hub at the center of the graph.

In nature, the coexistence of species with cyclic association is widely observed^31–37^. The well-mixed (global interaction) model never explains such a stable coexistence [see equation (2)]. To explain the biodiversity in RPS systems, plausible mechanisms for coexistence have been presented. A typical example is a local interaction: spatial distributions of species promote the coexistence of species^5,6,15,36,52^. In the present paper, we present another mechanism using the metapopulation model: the heterogeneity of the network promotes coexistence. In real ecosystems, the heterogeneous graph is more popular than the homogeneous one, since natural links are often disturbed by various obstructions.

## Electronic supplementary material

Supplementary Information

## References

[1] Gauze, G. F. *The struggle for existence*. (The Williams & Wilkins company, 1934).

[2] Tilman D (1994). Competition and Biodiversity in Spatially Structured Habitats. Ecology.

[3] Muko S, Iwasa Y (2000). Species Coexistence by Permanent Spatial Heterogeneity in a Lottery Model. Theor. Popul. Biol..

[4] Sugden AM (2001). Diversity Begets Diversity. Science (80-)..

[5] Tainaka K (1988). Lattice model for the Lotka-Volterra system. J. Phys. Soc. Japan.

[6] Tainaka K (1989). Stationary pattern of vortices or strings in biological systems: Lattice version of the Lotka-Volterra model. Phys. Rev. Lett..

[7] Tubay, J. M. *et al*. The paradox of enrichment in phytoplankton by induced competitive interactions. *Sci. Rep*. **3** (2013). 10.1038/srep02835 PMC3789149 24089056

[8] Chesson PL, Warner RR (1981). Environmental Variability Promotes Coexistence in Lottery Competitive Systems. Am. Nat..

[9] Bruno JF, Stachowicz JJ, Bertness MD (2003). Inclusion of facilitation into ecological theory. Trends Ecol. Evol..

[10] Janz N, Nylin S, Wahlberg N (2006). Diversity begets diversity: host expansions and the diversification of plant-feeding insects. BMC Evol. Biol..

[11] Tainaka K, Hashimoto T (2016). A Theory of Ratio Selection — Lattice Model for Obligate Mutualism. Open J. Ecol..

[12] Gatti RC, Hordijk W, Kauffman S (2017). Biodiversity is autocatalytic. Ecol. Modell..

[13] Itoh Y (1973). On a ruin problem with interaction. Ann. Inst. Stat. Math..

[14] Tainaka K (1993). Paradoxical effect in a three-candidate voter model. Phys. Lett. A.

[15] Nagatani, T., Sato, K., Ichinose, G. & Tainaka, K. Space promotes the coexistence of species: Effective medium approximation for rock-paper-scissors system. *Ecol. Modell*. **359** (2017).

[16] Nagatani T, Ichinose G, Tainaka K (2018). ichi. Traffic jams induce dynamical phase transition in spatial rock–paper–scissors game. Phys. A Stat. Mech. its Appl..

[17] Hashimoto T, Sato K, Ichinose G, Miyazaki R, Tainaka K (2017). Clustering Effect on the Dynamics in a Spatial Rock-Paper-Scissors System. J. Phys. Soc. Japan.

[18] Frachebourg L, Krapivsky PL, Ben-Naim E (1996). Segregation in a one-dimensional model of interacting species. Phys. Rev. Lett..

[19] Szolnoki A, Perc M (2016). Biodiversity in models of cyclic dominance is preserved by heterogeneity in site-specific invasion rates. Sci. Rep..

[20] Szolnoki A, Perc M (2016). Zealots tame oscillations in the spatial rock-paper-scissors game. Phys. Rev. E.

[21] Szolnoki, A. & Perc, M. Vortices determine the dynamics of biodiversity in cyclical interactions with protection spillovers. *New J. Phys*. **17** (2015).

[22] Levin SA (1974). Dispersion and Population Interactions. Am. Nat..

[23] Tainaka K, Itoh Y (2002). Patch dynamics based on Prisoner’s Dilemma game: superiority of golden rule. Ecol. Modell..

[24] Hanski, I., Gaggiotti, O. E. & ebrary, I. *Ecology*, *genetics*, *and evolution of metapopulations* (Burlington, MA: Elsevier, 2004).

[25] Reichenbach T, Mobilia M, Frey E (2007). Mobility promotes and jeopardizes biodiversity in rock-paper-scissors games. Nature.

[26] Claussen JC, Traulsen A (2008). Cyclic dominance and biodiversity in well-mixed populations. Phys. Rev. Lett..

[27] Peltomäki M, Alava M (2008). Three- and four-state rock-paper-scissors games with diffusion. Phys. Rev. E.

[28] Berr M, Reichenbach T, Schottenloher M, Frey E (2009). Zero-one survival behavior of cyclically competing species. Phys. Rev. Lett..

[29] Szolnoki A (2014). Cyclic dominance in evolutionary games: a review. J. R. Soc. Interface.

[30] Tainaka K, Fukazawa S (1992). Spatial pattern in a chemical reaction system: prey and predator in the position-fixed limit. J. Phys. Soc. Japan.

[31] Sinervo B, Lively CM (1996). The rock-paper-scissors game and the evolution of alternative male strategies. Nature.

[32] Burrows MT (1998). Modelling patch dynamics on rocky shores using deterministic cellular automata. Mar. Ecol. Prog. Ser..

[33] Buss LW (1980). Competitive intransitivity and size-frequency distributions of interacting populations. Proc. Natl. Acad. Sci..

[34] Paquin CE, Adams J (1983). Relative fitness can decrease in evolving asexual populations of S. cerevisiae. Nature.

[35] Durrett R, Levin SA (1994). Stochastic spatial models: a user’s guide to ecological applications. Philos. Trans. R. Soc. London. Ser. B Biol. Sci..

[36] Kerr B, Riley MA, Feldman MW, Bohannan BJM (2002). Local dispersal promotes biodiversity in a real-life game of rock-paper-scissors. Nature.

[37] Kirkup BC, Riley MA (2004). Antibiotic-mediated antagonism leads to a bacterial game of rock-paper-scissors *in vivo*. Nature.

[38] Sugiura K (2016). Population dynamics for freshwater species with cyclic relation. Far East J. Appl. Math..

[39] Szabó G, Fáth G (2007). Evolutionary games on graphs. Phys. Rep..

[40] Reichenbach T, Mobilia M, Frey E (2007). Noise and correlations in a spatial population model with cyclic competition. Phys. Rev. Lett..

[41] Izsák R, Szabó G, Szolnoki A (2004). Rock-scissors-paper game on regular small-world networks. J. Phys. A. Math. Gen..

[42] Masuda N, Konno N (2006). Networks with dispersed degrees save stable coexistence of species in cyclic competition. Phys. Rev. E - Stat. Nonlinear, Soft Matter Phys..

[43] Kitamura, K. *et al*. Asymmetrical effect of migration on a prey-predator model. *Phys. Lett. A* **357** (2006).

[44] Sato K, Hasegawa T, Morita S, Yoshimura J, Tainaka K (2015). Advantage or disadvantage of migration in a prey-predator system. Far East J. Appl. Math..

[45] Blasius B, Huppert A, Stone L (1999). Complex dynamics and phase synchronization in spatially extended ecological systems. Nature.

[46] Schwartz MK, Mills LS, McKelvey KS, Ruggiero LF, Allendorf FW (2002). DNA reveals high dispersal synchronizing the population dynamics of Canada lynx. Nature.

[47] Venkat S, Pleimling M (2010). Mobility and asymmetry effects in one-dimensional rock-paper-scissors games. Phys. Rev. E.

[48] Cheng H (2014). Mesoscopic interactions and species coexistence in evolutionary game dynamics of cyclic competitions. Sci. Rep..

[49] Horsthemke W, Lam K, Moore PK (2004). Network topology and Turing instabilities in small arrays of diffusively coupled reactors. Phys. Lett. A.

[50] Masuda, N., Porter, M. A. & Lambiotte, R. Random walks and diffusion on networks. *Phys. Rep*. 10.1016/j.physrep.2017.07.007 (2017).

[51] Juul J, Sneppen K, Mathiesen J (2013). Labyrinthine clustering in a spatial rock-paper-scissors ecosystem. Phys. Rev. E - Stat. Nonlinear, Soft Matter Phys..

[52] Itoh Y, Tainaka K (1994). Stochastic limit cycle with power-law spectrum. Phys. Lett. A.

