# Supplementary material for: Heterogeneous network promotes species coexistence: metapopulation model for rock-paper-scissors game
Source: Sci Rep. 2018 May 4;8:7094. doi: 10.1038/s41598-018-25353-4 (PMC5935761; doi:10.1038/s41598-018-25353-4)
Supplement: Supplementary file 1 — Supplementary Information [file 41598_2018_25353_MOESM1_ESM.doc]

**Supplementary Information**

**Title: Heterogeneous network promotes species coexistence: metapopulation model for rock-paper-scissors game**

**Authors: T. Nagatani1, G. Ichinose and K. Tainaka**

**--------------------------------------------------------------------------------------------------------**

**Basic Equations**

**1. Homogeneous network**

We consider the homogeneous network displayed in Fig. 1 (a). The densities of R, S, and P individuals in subpopulation (node) 1 are given by

$d\rho_{R,1}(t)/dt=[\frac{1}{2}\rho_{R,2}(t)+\frac{1}{2}\rho_{R,3}(t)-\rho_{R,1}(t)]+[a\rho_{R,1}(t)\rho_{S,1}(t)-c\rho_{P,1}(t)\rho_{R,1}(t)]$, (S1)

$d\rho_{S,1}(t)/dt=[\frac{1}{2}\rho_{S,2}(t)+\frac{1}{2}\rho_{S,3}(t)-\rho_{S,1}(t)]+[b\rho_{S,1}(t)\rho_{P,1}(t)-a\rho_{R,1}(t)\rho_{S,1}(t)]$, (S2)

$d\rho_{P,1}(t)/dt=[\frac{1}{2}\rho_{P,2}(t)+\frac{1}{2}\rho_{P,3}(t)-\rho_{P,1}(t)]+[c\rho_{P,1}(t)\rho_{R,1}(t)-b\rho_{S,1}(t)\rho_{P,1}(t)]$, (S3)

where the first and second terms on the right-hand side denote the migration and RPS game, respectively. Similarly, the densities of R, S, and P in both nodes 2 and 3 are given by

$d\rho_{R,2}(t)/dt=[\frac{1}{2}\rho_{R,1}(t)+\frac{1}{2}\rho_{R,3}(t)-\rho_{R,2}(t)]+[a\rho_{R,2}(t)\rho_{S,2}(t)-c\rho_{P,2}(t)\rho_{R,2}(t)]$, (S4)

$d\rho_{P,2}(t)/dt=[\frac{1}{2}\rho_{P,1}(t)+\frac{1}{2}\rho_{P,3}(t)-\rho_{P,2}(t)]+[c\rho_{P,2}(t)\rho_{R,2}(t)-b\rho_{S,2}(t)\rho_{P,2}(t)]$, (S5)

$d\rho_{S,2}(t)/dt=[\frac{1}{2}\rho_{S,1}(t)+\frac{1}{2}\rho_{S,3}(t)-\rho_{S,2}(t)]+[b\rho_{S,2}(t)\rho_{P,2}(t)-a\rho_{R,2}(t)\rho_{S,2}(t)]$, (S6)

$d\rho_{R,3}(t)/dt=[\frac{1}{2}\rho_{R,1}(t)+\frac{1}{2}\rho_{R,2}(t)-\rho_{R,3}(t)]+[a\rho_{R,3}(t)\rho_{S,3}(t)-c\rho_{P,3}(t)\rho_{R,3}(t)]$, (S7)

$d\rho_{P,3}(t)/dt=[\frac{1}{2}\rho_{P,1}(t)+\frac{1}{2}\rho_{P,2}(t)-\rho_{P,3}(t)]+[c\rho_{P,3}(t)\rho_{R,3}(t)-b\rho_{S,3}(t)\rho_{P,3}(t)]$, (S8)

$d\rho_{S,3}(t)/dt=[\frac{1}{2}\rho_{S,1}(t)+\frac{1}{2}\rho_{S,2}(t)-\rho_{S,3}(t)]+[b\rho_{S,3}(t)\rho_{P,3}(t)-a\rho_{R,3}(t)\rho_{S,3}(t)]$. (S9)

The steady-state (equilibrium) solution can be obtained by setting all the time derivatives to zero. The equilibrium densities are given by

$(\rho_{R,i,e},\rho_{P,i,e},\rho_{S,i,e})=(b/3(a+b+c),a/3(a+b+c),c/3(a+b+c))$ (S10)

for any node ($i=1,2,3$), where the suffix “e” denotes the equilibrium.

In particular, if $a=b=c=1$ (standard RPS game), we have

$\rho_{\alpha,\mathrm{i},e}=1/9$ (S11)

for any species $\alpha$ and node $i$ ($\alpha$=R,S,P and $i=1,2,3$). Numerical simulations reveal that the equilibrium densities [(S10) or (S11)] are not stable.

**2. Heterogeneous network**

Next, we consider the heterogeneous network displayed in Fig. 1 (b). The densities of R, S, P in nodes 1, 2, and 3 are given by

$d\rho_{R,1}(t)/dt=[\rho_{R,2}(t)+\rho_{R,3}(t)-\rho_{R,1}(t)]+[a\rho_{R,1}(t)\rho_{S,1}(t)-c\rho_{P,1}(t)\rho_{R,1}(t)]$, (S12)

$d\rho_{P,1}(t)/dt=[\rho_{P,2}(t)+\rho_{P,3}(t)-\rho_{P,1}(t)]+[c\rho_{P,1}(t)\rho_{R,1}(t)-b\rho_{S,1}(t)\rho_{P,1}(t)]$, (S13)

$d\rho_{S,1}(t)/dt=[\rho_{S,2}(t)+\rho_{S,3}(t)-\rho_{S,1}(t)]+[b\rho_{S,1}(t)\rho_{P,1}(t)-a\rho_{R,1}(t)\rho_{S,1}(t)]$, (S14)

$d\rho_{R,2}(t)/dt=[\frac{1}{2}\rho_{R,1}(t)-\rho_{R,2}(t)]+[a\rho_{R,2}(t)\rho_{S,2}(t)-c\rho_{P,2}(t)\rho_{R,2}(t)]$, (S15)

$d\rho_{P,2}(t)/dt=[\frac{1}{2}\rho_{P,1}(t)-\rho_{P,2}(t)]+[c\rho_{P,2}(t)\rho_{R,2}(t)-b\rho_{S,2}(t)\rho_{P,2}(t)]$, (S16)

$d\rho_{S,2}(t)/dt=[\frac{1}{2}\rho_{S,1}(t)-\rho_{S,2}(t)]+[b\rho_{S,2}(t)\rho_{P,2}(t)-a\rho_{R,2}(t)\rho_{S,2}(t)]$, (S17)

$d\rho_{R,3}(t)/dt=[\frac{1}{2}\rho_{R,1}(t)-\rho_{R,3}(t)]+[a\rho_{R,3}(t)\rho_{S,3}(t)-c\rho_{P,3}(t)\rho_{R,3}(t)]$, (S18)

$d\rho_{P,3}(t)/dt=[\frac{1}{2}\rho_{P,1}(t)-\rho_{P,3}(t)]+[c\rho_{P,3}(t)\rho_{R,3}(t)-b\rho_{S,3}(t)\rho_{P,3}(t)]$, (S19)

$d\rho_{S,3}(t)/dt=[\frac{1}{2}\rho_{S,1}(t)-\rho_{S,3}(t)]+[b\rho_{S,3}(t)\rho_{P,3}(t)-a\rho_{R,3}(t)\rho_{S,3}(t)]$. (S20)

The equilibrium densities depend on nodes. When $i=1$ (“hub”), we get

$(\rho_{R,1,e},\rho_{S,1,e},\rho_{P,1,e})=(b/2(a+b+c),c/2(a+b+c),a/2(a+b+c))$. (S21)

When the node is not hub, the equilibrium densities take the same values for both nodes 2 and 3:

$\begin{matrix} (\rho_{R,2,e},\rho_{S,2,e},\rho_{P,2,e})=(\rho_{R,3,e},\rho_{S,3,e},\rho_{P,3,e}) \\ =(b/4(a+b+c),c/4(a+b+c),a/4(a+b+c)) \end{matrix}$ , (S22)

Hence, the densities in node 2 (3) are just half, compared to those in node 1. In particular, if $a=b=c=1$, we have

$\rho_{\alpha,1,e}=1/6$, $\rho_{\alpha,2,e}=\rho_{\alpha,3,e}=1/12$. (S23)

Numerical simulations show that the above densities are stable. The values of the final equilibrium point are consistent with those obtained by numerical simulations. The total density in node $i$ is defined by$\rho_{i}= \rho_{Ri}(t)+\rho_{Pi}(t)+\rho_{Si}(t)$. Thus, we get

$\rho_{1,e}=1/2$, $\rho_{2,e}=\rho_{3,e}=1/4$. (S24)

The relation (S24) means that individuals gather in node 1 (hub) by random migration.
